# Modulation of the Proteostasis Machinery to Overcome Stress Caused by Diminished Levels of t^6^A-Modified tRNAs in *Drosophila*

**DOI:** 10.3390/biom7010025

**Published:** 2017-03-06

**Authors:** Diego Rojas-Benítez, Cristián Eggers, Alvaro Glavic

**Affiliations:** Centro de Regulación del Genoma, Facultad de Ciencias, Universidad de Chile, Las Palmeras 3425, Ñuñoa, Santiago 7800024, Chile; diegorojasb@ug.uchile.cl (D.R.-B.); ceggersa@gmail.com (C.E.)

**Keywords:** tRNA post-transcriptional modification, *N*^6^-threonylcarbamoyladenosine, unfolded protein response

## Abstract

Transfer RNAs (tRNAs) harbor a subset of post-transcriptional modifications required for structural stability or decoding function. *N*^6^-threonylcarbamoyladenosine (t^6^A) is a universally conserved modification found at position 37 in tRNA that pair A-starting codons (ANN) and is required for proper translation initiation and to prevent frame shift during elongation. In its absence, the synthesis of aberrant proteins is likely, evidenced by the formation of protein aggregates. In this work, our aim was to study the relationship between t^6^A-modified tRNAs and protein synthesis homeostasis machinery using *Drosophila melanogaster*. We used the Gal4/UAS system to knockdown genes required for t^6^A synthesis in a tissue and time specific manner and in vivo reporters of unfolded protein response (UPR) activation. Our results suggest that t^6^A-modified tRNAs, synthetized by the threonyl-carbamoyl transferase complex (TCTC), are required for organismal growth and imaginal cell survival, and is most likely to support proper protein synthesis.

## 1. Introduction

**Figure 1:**
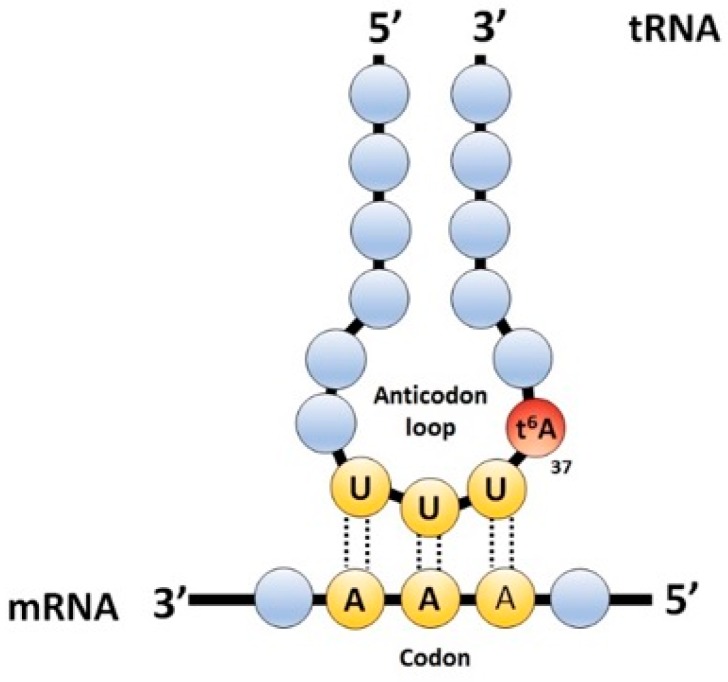
*N*^6^-threonylcarbamoyladenosine (t^6^A) is harbored in position 37 of A-starting codons (ANN)-pairing transfer RNAs (tRNAs). The schematic representation of the anticodon loop of an ANN-pairing tRNA: t^6^A at position 37 is represented as a red full circle; the anticodon is represented in yellow; and the rest of the tRNA in light blue. Messenger RNA (mRNA) is represented at the bottom in light blue circles and the codon in yellow. Codon-anticodon interactions are depicted with dotted lines, and are stabilized by t^6^A, thus preventing intra-loop bonding between positions U33 and A37.

Transfer RNAs (tRNAs) are structured and stable nucleic acids, transcribed by RNA Polymerase III in eukaryotes. During protein synthesis, they act together with ribosomes as decoders of information contained in messenger RNAs (mRNAs), recognizing successive codons to add specific amino acids to nascent polypeptide chains [1]. After transcription, tRNAs are extensively processed and modified. A distinctive feature of tRNAs is the high level of post-transcriptional modification that is held, each one presents a subset of over 90 known modifications [2]. Depending on their position, they play structural roles [3] or are required for proper decoding activity stabilizing cognate base pairing or expediting wobble base pairing [4], thus increasing decoding capability [5]. Additionally, these kinds of modification prevent frame-shift [6], ensuring correct translation. Position 37 (adjacent to the anticodon) is frequently modified, even more so than the wobble position [7]. The most common modification present in purines at position 37 are *N*^6^-isopentenyladenosine (i^6^A), *N*^6^-isopenetyl-2-thiomethy-ladenosine (mS^2^i^6^A), 1-methylguanosine (m^1^G) and *N*^6^-threonylcarbamoyladenosine (t^6^A). The last is present in tRNAs that pair A-starting codons (ANN) (Figure 1) [2]. This modification is universally conserved and has a paramount role for tRNA decoding function, as has been shown in yeast [8,9], archea [10], bacteria [11], and, recently by our laboratory [12] and others [13], in *Drosophila*. This modification was identified over 40 years ago [14,15,16]; however, the enzymes that synthetize it were only recently identified [8,9]. Tcs1 (Yrdc) or Tcs2 (Sua5) catalyzes the formation of a l-threonyl-cabamoyl-AMP (TC-AMP) intermediate from bicarbonate, threonine and ATP. Next, the threonyl-carbamoyl transferase complex (TCTC, previously named KEOPS/EKC (kinase, endopeptidase and other proteins of small size/endopeptidase-like and kinase associated to transcribed chromatin)) is responsible for the last step of the reaction, transferring the TC-group to A37 in substrate tRNA [17]. This synthetic pathway is universally conserved [18]. In silico analyses have found Tcs2 (CG33786) and the TCTC subunit homologues in *Drosophila*; TCTC complex is composed of Tcs3 (Kae1), the catalytic subunit that physically interacts with Tcs5 (Prpk) and has regulatory functions over Tcs3; while Tsc6 (Pcc1) allows dimerization, two more subunits compose the yeast TCTC complex, Tcs4 (Qri7) and Tcs8 (Gon7), but there are no counterparts in *Drosophila*. Mutations in either the gene coding for TCTC subunits or Tcs2 eliminate t^6^A in tRNA and cause strong slow-growth phenotype in yeast [8], as well as cell and organismal size reductions in *Drosophila* [12,19]. At the molecular level, yeast lacking t^6^A present erroneous protein synthesis initiation at non-AUG codons and frameshifts during elongation [8], making the synthesis of misfolded and unfolded proteins very likely, as evidenced by the formation of protein aggregates [20]. Upon *Tcs3* knockdown in *Drosophila*, a collection of signaling pathways, called the unfolded protein response (UPR), is activated in order to overcome stress caused by aberrant proteins in the lumen of the endoplasmic reticulum (ER), thus reestablishing protein homeostasis. Nonetheless, its chronic activation leads to apoptosis [21,22]. Chronic activation of the UPR is a likely explanation for the phenotypes observed upon loss of function of the TCTC subunits. In this work, our aim was to establish functional relationships between t^6^A-modified tRNAs and the protein synthesis homeostasis machinery in *Drosophila*. We show that a loss of function in the t^6^A synthetic machinery causes apoptosis and the UPR activation in imaginal cells, indicating that the role of t^6^A-modified tRNAs is to support correct protein synthesis.

## 2. Results

We have previously shown that *Drosophila* mutants for *tcs3* have extremely low levels of t^6^A-modified tRNAs. The phenotype of these mutants is too severe to establish functional relationships or underlying causes of phenotypes. In order to overcome this, we took a different approach using the binary Gal4/UAS system [23], which was originally created to study gene expression and was adapted to knockdown. Driver lines were used to express the yeast transcription factor Gal4 under a specific promoter. By itself, this does not present effects on cells, since it must bind to an upstream activating sequence (UAS) region to activate transcription. There is a plethora of flies with an UAS region upstream to a desired sequence to be expressed, either a coding sequence (i.e., green fluorescent protein (GFP)) or an inverted repeated (IR) to transcribe a RNA hairpin to activate specific RNA interference (RNAi) [24]. Gal4 and UAS lines were mated to knockdown *Tcs2* and TCTC components either ubiquitously, or in a tissue-specific manner. A list of driver lines (Table 1) and UAS lines (Table 2) is provided.

### 2.1. t^6^A Synthetic Machinery is Required for Larval Growth

**Figure 2:**
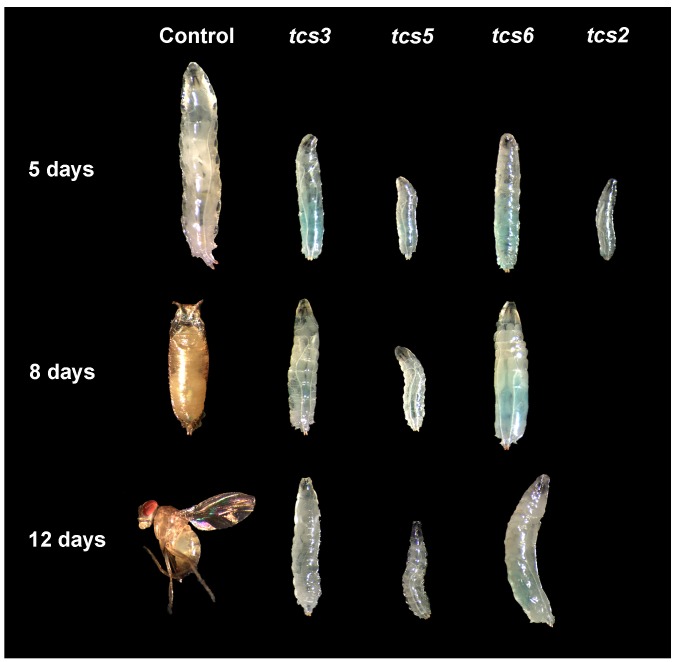
Ubiquitous knockdown of t^6^A modification system results in reduced larval size. Ubiquitous knock down of components of the threonyl-carbamoyl transferase complex (TCTC) and *tcs2* was achieved using the Gal4/UAS system. For this, the *tub* > Gal4 driver was used and animal size was evaluated at three different time points. The figure shows the representative images of the individuals depicted at different times after egg laying (AEL).

To shed light on t^6^A-modified tRNA function in *Drosophila* using the *tubulin* driver (*tub* > Gal4), we knocked-down genes required for t^6^A synthesis using different validated UAS-IR constructs [25,26,27]. Reduced larval size was observed by Day 5 after egg laying (AEL) (Figure 2). By Day 8, the AEL control individuals had pupariated, but when TCTC components or *tcs2* were silenced, smaller larvae were observed with no pupa features. In the case of the *tcs2* knockdown, larvae were small by Day 5 and died by Day 8 AEL. In the controls, by Day 12 adults are observed, while in the loss of function only larvae were present. These results, which are consistent with previous data, suggest that all silenced genes, namely t^6^A-modified tRNAs, are required for animal growth.

### 2.2. *Tcs3* is Required for Imaginal Cell Survival

**Figure 3:**
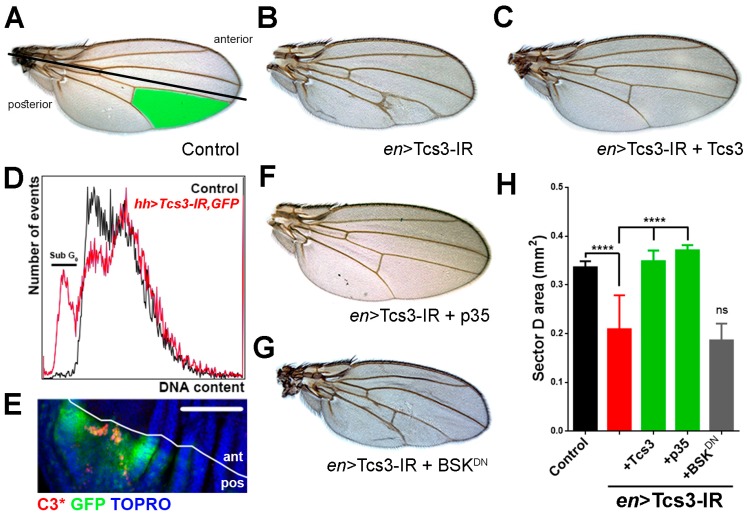
*tcs3* knockdown causes apoptosis in imaginal cells. (**A**) Using the Gal4/UAS system *tcs3* was knocked-down in the wing disc posterior compartment (*engrailed* driver, *en* > Gal4; bottom part of the wing where the boundary between the anterior and posterior compartments is indicated with a black line). Representative pictures of the observed phenotypes are shown. Control (*en* > Gal4/+); (**B**) *tcs3* knock down (*en* > Tcs3-IR); (**C**) co-expression of Tcs3 and Tsc3-IR; (**D**) *hedgehog* driver (*hh* > Gal4) was used to knock down *tcs3* and express GFP in the wing posterior compartment. Wing discs were disaggregated and cells analyzed by flow cytometry. Control cells (GFP-, anterior compartment) and GFP+ (*hh* > Tcs3-IR, GFP) were analyzed for DNA content (DRAQ5). The sub G1 population is indicated by a black line over the curve; (**E**) cleaved Caspase-3 (C3*) was detected by immunofluorescence in wing discs in which tcs3 was silenced in the GFP expressing domain using the *hedgehog* driver. A white line indicates the boundary of the anterior (ant) and posterior (pos) compartments; bar 100 µm; (**F**) Simultaneous *tcs3* knockdown and p35 expression or (**G**) BSK dominant negative (BSK^DN^) expression. (**H**) Quantification of the area of sector D in the wings of these animals (green section in control) (*n* = 15, ANOVA, *p* < 0.005).

Ubiquitous knockdown of *tcs2* and the TCTC subunits caused a severe phenotype, a condition for which underlying causes are difficult to establish. To overcome this, we made a tissue-specific knockdown. The *Drosophila* adult structures arise from groups of highly proliferative cells called imaginal discs where wings are formed from wing imaginal discs [28]. We silenced *tcs3*, the catalytic subunit of the TCTC, in the posterior compartment of the developing wing imaginal discs using the *engrailed* driver (*en* > Gal4) [29], so that any effect on wing development could be later analyzed in the adult structures. This allowed us to evaluate functional relationships between genes using a morphological trait in wings (area of sector D) (Figure 3A, green-colored area). A collapse between veins IV and V (Figure 3B,H) was regularly observed when *tcs3* was silenced. In order to test knockdown specificity, we crossed flies in which *tcs3* was knocked-down with null mutants for *tcs3*, generating a heterozygous mutant background in which the sector D area was further reduced (data not shown). In addition, we simultaneously silenced *tcs3* and expressed the *Tcs3* coding sequence, a complete reversion of the phenotype was observed (Figure 3C,H). These results showed that the observed phenotype was specific to the *tcs3* knockdown.

As the observed veins were malformed, we wondered if this phenotype was produced by apoptosis occurring in the wing imaginal cells. To investigate this, we simultaneously knocked-down *tcs3* and expressed GFP in the posterior compartment of the wing imaginal discs using the *hedgehog* driver (*hh* > Gal4), and analyzed the DNA content in the control (GFP-) and GFP-expressing/*tsc3* knockdown cells by flow cytometry (Figure 3D). In cells where *tcs3* was knocked-down, we detected a reduction in the G_0_/G_1_ cell population and a new population of cells in the sub-G_0_/G_1_ region, which exhibited a deficit in DNA content, a feature consistent with apoptotic cells [30]. In order to confirm this, we detected cleaved Caspase-3 in the wing imaginal discs by immunohistochemistry. Positive patches for cleaved Caspase-3 were present only in the posterior compartment, indicating that apoptosis was induced only in the region where *tcs3* was silenced (Figure 3E). In order to prove whether apoptosis was the underlying cause of the phenotype upon *tcs3* knockdown, we simultaneously overexpressed the anti-apoptotic protein p35 [31,32] where a reversion of the phenotype similar to the expressed *Tcs3* was observed (Figure 3F,H). In contrast, the overexpression of a dominant negative form of Basket (BSK^DN^), the *Drosophila* orthologue of c-Jun N-terminal kinase (JNK) required apoptosis activation in several stressful conditions [33], did not rescue the phenotype (Figure 3E,H). Thus, our results showed that *Tcs3* is required for the survival of imaginal cells and suggest that apoptosis of imaginal cells, independent of BSK, is the underlying cause of the phenotype.

### 2.3. Activation of UPR upon Silencing of t^6^A Synthetic Machinery

**Figure 4:**
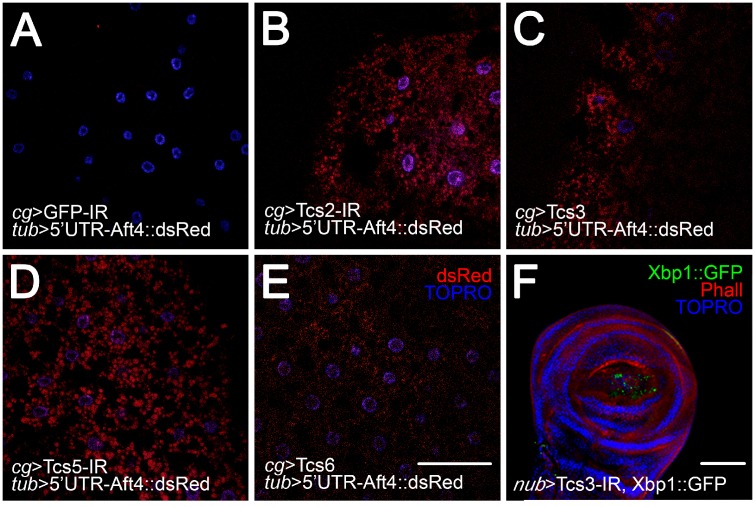
Unfolded protein response (UPR) is activated in larval and imaginal cells deficient of t^6^A modified tRNAs. In vivo UPR activation reporters were used. (**A**–**E**) Construct in which the 5’UTR of Atf4 mRNA was fused with dsRed and expressed ubiquitously; when the PERK/Atf4 branch of the UPR is activated, dsRed is translated. In this reporter background, t^6^A synthetic machinery was knocked-down exclusively in the fat body using *collagen type IV* driver (*cg* > Gal4). Nuclei were stained with TO-PRO3. Bar 100 µm; (**F**) *tcs3* was knocked-down using the *nubbin* > Gal4 driver, simultaneously an Ire1 branch UPR reporter was expressed. Upon UPR activation, the Xbp-1::GFP message was spliced and the fusion protein synthetized. F-actin was stained with phalloidin (Phall) and nuclei with TO-PRO3 (TOPRO). Bar 100 µm.

**Figure 5:**
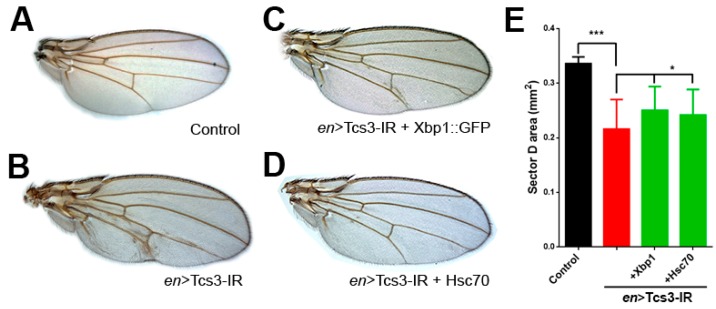
Enhancement of protein folding capacity of cells partially reverted wing phenotype. (**A**) Control wings (*en* > Gal4/+), using the *en* > Gal4 driver we simultaneously knocked-down *tcs3* (**B**) and overexpressed (**C**) Xbp1::GFP or (**D**) Hsc70. (**E**) Quantification of the area of sector D (*n* = 15, ANOVA, *p* < 0.005).

t^6^A is a structural feature that allows the correct recognition of ANN codons by its cognate tRNA. It has been suggested that its absence causes incorrect protein synthesis initiation and enhances the chance of frameshifting during translation in yeast [8]. Under these conditions, the synthesis of misfolded and unfolded proteins is very likely, as evidenced by the formation of protein aggregates [20]. When unfolded proteins populate the lumen of the ER it leads to ER stress, which activates the UPR, a collection of signaling pathways aiming to reestablish protein synthesis homeostasis [34] if this is not accomplished, apoptosis is induced [35]. In previous studies, we showed that *tcs3* or *tcs5* knockdown activated the UPR [21]. This analysis was extended to the complete set of t^6^A synthetic machinery genes in non-proliferative cells, the fat body (Figure 4A–E) and evaluated the UPR induction using an in vivo reporter of PERK/Atf4 activation. Atf4 mRNA translation control occurs due to several small upstream open reading frames (uORFs) in the 5’ UTR. The last one overlaps Atf4 coding sequence in a different reading frame, therefore inhibiting its translation in the unstressed cells where upon ER stress, the main reading frame is translated [22]. In the reporter, Atf4 5’UTR is fused to dsRed ORF, then in stressed cells dsRed will be translated [36]. In control animals, no dsRed signal was detected (Figure 4A); however, when *tcs2* (Figure 4B) or TCTC subunits were silenced (Figure 4C–E), a signal was detected, indicating that the UPR is active via the PERK/ATF4 pathway, confirming and extending our previous results for *tcs3* and *tcs5* [21]. In addition, we wondered whether the UPR activation upon *tcs3* silencing also took place in the imaginal cells (proliferative cells). In order to prove this, we used the *nubbin* driver (*nub* > Gal4) to knockdown *tcs3* and simultaneously express a different in vivo UPR activation reporter, in which a Xbp-1::GFP fusion protein was translated only after non-canonical splicing of Xbp-1::GFP mRNA is induced upon Ire1 activation by ER stress [37]. Upon *tcs3* knockdown, we detected GFP in the *nub* > Gal4 territory (Figure 4F), indicating the UPR was active in these cells. We wondered if chronic ER stress and the UPR activation were the underlying causes of the observed phenotype in wings. To address this, we simultaneously silenced *tcs3* and overexpressed Xbp-1::GFP (Figure 5C) or Hsc70 (Figure 5D), the *Drosophila* orthologue of BIP/GRP78 (the main ER chaperone) [38], where both partially reverted the phenotype (Figure 5E). These results show that Tcs3 is required for organismal growth and the survival of imaginal cells in *Drosophila*, and most likely supports proper protein synthesis.

## 3. Discussion

Nucleic acids in cells are post-transcriptionally modified, a phenomenon known for several decades. In RNA, over 90 modifications have been identified, most of them found in tRNAs [39]. Modifications present in the body of tRNA are usually relevant for folding and stability [40,41], while the ones present in the anticodon wobble position 34 and base 37, adjacent to the anticodon are required for proper decoding capabilities. Some of these modifications such as *N*^6^-threonylcarbamoyladenosine (t^6^A) are universally conserved and its synthesis requires a multi-enzymatic pathway, which is present in all domains of life, suggesting that it is part of the minimal translation machinery [42] and could be considered as a primordial modification [43]. The study of this modification would allow us to understand not only how the translation machinery works, but also how it has evolved as this modification would have eased the evolution of highly-accurate translation systems [44]. Intensive study of tRNA modification using metazoan models is imperative as tRNA modification have been linked to numerous human pathologies (reviewed in references [45,46]).

Our group [12] and others [13] have observed that *tcs3* mutants lacked imaginal discs and larval tissues were smaller, indicating that different cell types could have particular requirements for t^6^A-modified tRNAs; consistently *Drosophila tcs3* [47] and human *TCS3*/OSGEP [48] are differentially expressed in different tissues, supporting a tissue-specific demand. Accordingly, silencing the TCTC components in differentiated *Drosophila* photoreceptors did not cause any phenotype [13], suggesting that differentiated cells have a low demand for t^6^A-modified tRNAs; in contrast, highly proliferative cells such as the ones that the wing imaginal discs in *Drosophila* are composed, seem to have a high demand for these tRNAs. There could be a cell cycle-dependent requirement of t^6^A-modified tRNAs even in the same cell type, as we observed only G_0_/G_1_ cells die by apoptosis, which is consistent with the severe target of rapamycin (TOR) inhibition in *tcs3* mutants [12] as its activity promotes G_1_/S progression [49].

One could propose that the absence of t^6^A has a global and disastrous effect on translation; however, this appears not to be the case. Even with twice the erroneous translation start in non-AUG codons and doubling the translational ambiguities, only a subset of genes was affected, suggesting that the pleiotropic phenotype observed was caused not by a global translational problem, but depended on codon-specific defects [20]. Modified bases in tRNAs modulate protein synthesis [50]. Codon bias contributes to establish protein level in order to adjust production rate of a particular proteins to changing requirements (e.g., stressful conditions, cell cycle progression, differentiation, etc.), and tRNA modifications are regulated, resulting in preferential translation of mRNAs coding the required proteins [51,52]. Different cell types, each one having particular translational requirements, would be differentially affected, as each will have particular codon biases and requirements for modified tRNAs. For instance, imaginal cells die when t^6^A-modified tRNAs levels are diminished; on the contrary, larval tissue deals with this by lowering their anabolic rate and survive. Ribosome profiling combined with proteomics from different tissues would help to elucidate this in *Drosophila*.

As imaginal cells were more affected than other cell types, we silenced *tcs3* in the posterior wing compartment using the Gal4 driver *engrailed* and observed a reduction in the sector D area, a phenotype we demonstrated was caused specifically by *tcs3* knockdown. This change in morphological trait allowed us to establish functional relationships. The reduction in sector D was rescued by blocking apoptosis with p35, an anti-apoptotic protein [31]. We also asked what activated apoptosis when t^6^A-modified tRNAs levels were diminished. One possible explanation for the observed phenotype is the chronic activation of the UPR. Silencing either component of the TCTC activated the UPR in larval and imaginal tissue. We noticed that when cellular folding capacity was enhanced—either by overexpression of Xbp1 or Hsc70—a partial rescue of the phenotype was observed. This indicates that at least in part, the phenotype was caused by aberrant protein overload at the ER. Another component of the phenotype that was not explored further, was the observation of differential ORF selection by the levels of t^6^A-modified tRNAs. A fact that, independently from the UPR activation, could explain the Atf4-5’UTR::dsRed reporter activation. Nonetheless, our previous results, using a different reporter, showed that the UPR was active when *tcs3* or *tcs5* was silenced [21]. Thus, diminished levels of t^6^A-modified tRNAs would have at least two different impacts on protein synthesis: making it error-prone and to favor or inhibit specific ORF translation, creating a disturbance in protein synthesis homeostasis.

A series of unanswered questions remain about t^6^A-modified tRNAs and its relationship with protein synthesis and other cellular processes, for instance, in previous studies we showed that t^6^A-modified tRNAs were related, by an unknown mechanism, to TOR activity [12] and endocytosis [19]. Other modifications present in the anticodon loop, such as methoxycarbonylmethyl-2-thiouridine (mcm^5^s^2^U) also affect translation efficiency and compromise TOR activity [53] and activate GCN4-dependent transcription [54], common features that have been observed in mutant yeast for TCTC subunits [8,12,19]. Therefore, these observations suggest that there are unknown molecular mechanisms that link tRNA modification to stress-response mechanisms and other central components of cellular physiology. How *tcs3* expression and more importantly, how tRNA modifications are regulated in different cell types or through cell cycle are questions that require further research.

## 4. Materials and Methods

### 4.1. Fly Husbandry and Fly Stocks

Animals were raised at low density at 25 °C on standard meal containing wheat flour (50 g/L); fresh yeast (100 g/L); agar-agar (11 g/L); dextrose monohydrate (80 g/L); propionic acid (6 mL/L); and Nipagin (1.56 g/L). Stocks were obtained from the Bloomington Drosophila Stock Center (BDSC) and the Vienna Drosophila Resource Center (VDRC) [25]. The Atf4 5’UTR::dsRed reporter is described in reference [36] and Xpb1::GFP is described in reference [37].

### 4.2. Morphological, Morphometric and Statistical Analysis

Adults of the corresponding genotypes were crossed for 24 h in vials with standard meal, then withdrawn and incubated for the indicated periods of time. Pictures of the larvae were taken with a Nikon SMZ800 stereoscope (Tokyo, Japan) using a Micrometrics 519CU OM camera and Micrometrics SE Premium 4 software (Unitron, New York, NY, USA). *Drosophila* wings were mounted in a 1:1 mixture of lactic acid/ethanol as described in reference [19] and photographed under an Olympus BX51 microscope (Tokyo, Japan) using a Moticam 2500 digital camera (Motic. XiangAn, Xiamen, China). The wing area was measured using Adobe Photoshop CS5 Extended (Adobe, San José, CA, USA). All data presented are mean ± standard deviation (s.d.) and were subjected to a one-way ANOVA test using GraphPad Prism 6 (GraphPad Software, La Jolla, CA, USA). *p* values lower than 0.05 were considered to be significant, unless otherwise indicated.

### 4.3. Immunofluorescence

Larvae were dissected and fixed as described in reference [56]. Confocal images were captured using a Zeiss LSM510 Meta confocal microscope (Oberkochen, Germany). Nuclei were stained with TO-PRO-3 (1:200, phalloidin (1:200, Invitrogen, Carlsbad, CA, USA) and cleaved Caspase-3 (1:200 Cell Signaling, Danvers, MA, USA).

### 4.4. Flow Cytometry

Larvae were dissected in the early third stage and wing imaginal discs were transferred to microtubes with 500 µL of Trypsin/EDTA (Sigma, St. Louis, MO, USA) and 10 µM DRAQ5 (Biostatus, Loughborough, UK). For disaggregation, imaginal discs were incubated a 25 °C with constant shaking for 2 h, and, to stop digestion, fetal bovine serum was added to a final concentration of 2%. Cells were filtered through a 0.35 µm mesh (Nythal, Heiden, Switzerland) and immediately analyzed in a FACSCanto flow cytometer (BD, Franklin Lakes, NJ, USA). Data were analyzed using FlowJo (TreeStar, Ashland, OR, USA).

## 5. Conclusions

t^6^A-modified tRNAs are required in *Drosophila melanogaster* for correct protein synthesis in order to support organismal growth and cell survival of imaginal cells.

## Figures and Tables

**Table 1:** List of Gal4 lines used.

Name	Expression Pattern	Reference
*tubulin* (*tub* > Gal4)	Ubiquitous	BDSC (5138)
*engrailed* (*en* > Gal4)	Posterior compartment	BDSC (1973)
*hedgehog* (*hh* > Gal4)	Posterior compartment	Mullor et al. [55]
*collagen type IV* (*cg* > Gal)	Fat body (larval tissue)	BDSC (7011)
*nubbin* (*nub* > Gal4)	Wing pouch (imaginal disc)	BDSC (42699)

BDSC: Bloomington Drosophila Stock Center.

**Table 2:** List of upstream activating sequence (UAS) lines used.

Name	Utility	Reference
UAS-Tcs2 IR	*tcs2* knockdown	VDRC (dna13368)
UAS-Tcs3 IR	*tcs3* knockdown	VDRC (106250)
UAS-Tcs5 IR	*tcs5* knockdown	VDRC (dna7059)
UAS-Tcs6 IR	*tcs6* knockdown	VDRC (4371)
UAS-GFP	GFP expression	BDSC (5137)
UAS-Tcs3	Tcs3 expression	Rojas-Benitez et al. [12]
UAS-p35	p35 expression	BDSC (5072)
UAS-BSK^DN^	Dominant negative BSK expression	BDSC (6409)
GFP-IR	GFP knockdown	BDSC (9331)
tub > Atf4 5’UTR::dsRed	UPR activation reporter	Kang et al. [36]
UAS-Xbp1::GFP	UPR activation reporter	Sone et al. [37]
UAS-Hsc70	Hsc70 expression	BDSC (5843)

VDRC: Vienna Drosophila Resource Center; BDSC: Bloomington Drosophila Stock Center.
